# Complete Genome Sequence of Veillonella nakazawae JCM 33966^T^ (=CCUG 74597^T^), Isolated from the Oral Cavity of Japanese Children

**DOI:** 10.1128/MRA.00279-21

**Published:** 2021-04-29

**Authors:** Izumi Mashima, Futoshi Nakazawa, Riyoko Tamai, Yusuke Kiyoura

**Affiliations:** a Department of Oral Medical Science, School of Dentistry, Ohu University, Koriyama, Fukushima, Japan; b Department of Oral Biology, Faculty of Dentistry, Universitas Indonesia, Jakarta, Indonesia; University of Rochester School of Medicine and Dentistry

## Abstract

We report the complete genome sequence of Veillonella nakazawae JCM 33966^T^ (=CCUG 74597^T^). This bacterium is a member of the oral *Veillonella* and has the potential to be anticariogenic as an oral probiotic seed.

## ANNOUNCEMENT

The genus *Veillonella* consists of small, strictly anaerobic, Gram-negative cocci that can be isolated from the oral cavity and intestinal tract of humans and animals. The bacteria gain energy from the utilization of short-chain organic acids, especially lactate, instead of using carbohydrates or amino acids and subsequently produce acetate and propionate (1–3). Recent reports have shown that the gut bacterium Veillonella atypica can improve the running efficiency of the host by converting the lactate produced during exercise into propionate, thus enhancing athletic performance (4). Therefore, members of the genus *Veillonella* are attractive for use as natural probiotic seeds and need further research.

Veillonella nakazawae was isolated from the oral cavity of Japanese children and was established as a novel *Veillonella* species in 2020 (5). At present, 8 species from the genus *Veillonella* have been isolated from the human oral cavity, and they are called oral *Veillonella* (5–11). These 8 species have shown lactate consumption ability (5–11). As lactate causes dental caries, it has been suggested that oral *Veillonella* may be anticariogenic (12). However, the anticariogenic mechanisms or functions of these species have not been clarified. Therefore, to promote the study of the mechanisms underlying the functioning of oral *Veillonella*, we determined the complete genome sequence of *V. nakazawae* JCM 33966^T^ (=CCUG 74597^T^), a *V. nakazawae* type strain (5).

Towards this end, *V. nakazawae* JCM 33966^T^ (=CCUG 74597^T^) was grown on Bacto brain heart infusion (BHI) medium (Difco Laboratories, BD) supplemented with 5% (vol/vol) defibrinated sheep blood for 2 days at 37°C under anaerobic conditions (N_2_, 80%; H_2_, 10%; CO_2_, 10%). Genomic DNA was extracted from this strain using the phenol-chloroform extraction and ethanol precipitation procedures (13). The quality of the genomic DNA obtained was checked using the Qubit 4 fluorometer (Thermo Fisher Scientific, MA, USA). The same genomic DNA was used for both long- and short-read sequencing. All DNA sequencing was performed and *de novo* assembly was generated by the Taniguchi Dental Clinic/Oral Microbiome Center (Kagawa, Japan) as per the manufacturer’s instructions.

For long-read sequencing, a DNA library was prepared using a native barcoding expansion kit (EXP-NBD104; barcodes 1 to 12; Oxford Nanopore, Oxford, UK) and ligation sequencing kit (SQK-LSK108; Oxford Nanopore) without DNA sharing according to the manufacturer’s instructions and was subsequently sequenced using the GridION X5 genome sequencing system with an R9.4.1 flow cell (FLO-MIN106). The long-read sequences were base called using Guppy v.3.6.0 (Oxford Nanopore), and the estimated *N*_50_ value was 10.9 kbp. After quality trimming (average Phred quality value of >10.0, short reads of >1,000 bp, and adaptor sequences) using NanoFilt v.2.7.1 software (14), a total of 40,817 reads were generated. Concurrently, for short-read sequencing, libraries were prepared for Illumina sequencing using a Nextera DNA Flex library prep kit (Illumina, San Diego, CA, USA), followed by sequencing using the MiSeq platform (Illumina). The raw sequencing data were processed using FASTQ v.0.20.1 (15) for trimming the adaptors, low-quality data (average Phred quality value of >30.0), and low sequencing data (short reads of >10 bp), yielding 1,825,183 short reads with an average length of 149.2 bp.

The remaining high-quality reads were then assembled *de novo* using the Unicycler v.0.4.8 software (16) and were visualized using Bandage v.0.8.1 software (17) to confirm a closed circular sequence. All software was operated using default settings and parameters unless otherwise specified. The final chromosome sequence was 2,097,818 bp (G+C content, 38.7%), and the final coverage of the genome was 259.8×.

The final chromosome was annotated using the DDBJ Fast Annotation and Submission Tool (DFAST) v.1.2.7.0 (https://dfast.nig.ac.jp/) with default parameters (18). The chromosome contained 1,925 coding DNA sequences (CDSs), 12 rRNAs, 48 tRNAs, and 1 CRISPR.

With the addition of the annotated genome sequence of *V. nakazawae* described here, the genome sequences of 8 species of oral *Veillonella* are now available to the public. In addition, the results of a genome comparison of *V. nakazawae* JCM 33966^T^ (=CCUG 74597^T^) against reported type strains of the genus *Veillonella* were reported in our recent study (5). The data obtained will help clarify the anticariogenic mechanisms or functions of *Veillonella* in future studies.

### Data availability.

The raw sequence data were deposited in the DDBJ Sequence Read Archive (SRA), and the complete genome sequence was deposited in DDBJ/GenBank/EMBL under the accession numbers DRA009432 and AP022321, respectively. The versions in this paper are the first versions reported. These accession numbers were originally published by Mashima et al. (5).
